# Reclassification of genetic variants in children with long QT syndrome

**DOI:** 10.1002/mgg3.1300

**Published:** 2020-05-08

**Authors:** Dominik S. Westphal, Tobias Burkard, Alexander Moscu‐Gregor, Roman Gebauer, Gabriele Hessling, Cordula M. Wolf

**Affiliations:** ^1^ Institute of Human Genetics Technical University of Munich Munich Germany; ^2^ Institute of Human Genetics Helmholtz Zentrum Munich Neuherberg Germany; ^3^ Department of Congenital Heart Disease and Pediatric Cardiology German Heart Center Munich, Technical University of Munich Munich Germany; ^4^ Center for Human Genetics and Laboratory Diagnostics Martinsried Germany; ^5^ Department of Pediatric Cardiology Heart Center Leipzig, University of Leipzig Leipzig Germany

**Keywords:** ACMG, cardiogenetics, long QT syndrome, Schwartz score, variant classification

## Abstract

**Background:**

Genes encoding cardiac ion channels or regulating proteins have been associated with the inherited form of long QT syndrome (LQTS). Complex pathophysiology and missing functional studies, however, often bedevil variant interpretation and classification. We aimed to evaluate the rate of change in variant classification based on current interpretation standards and dependent on clinical findings.

**Methods:**

Medical charts of children with a molecular genetic diagnosis of LQTS presenting at our centers were retrospectively reviewed. Reinterpretation of originally reported variants in genes associated with LQTS was performed based on current knowledge (March 2019) and according to the “Standards and Guidelines for the Interpretation of Sequence Variants” by the ACMG 2015.

**Results:**

About 84 distinct (likely) pathogenic variants identified in 127 patients were reinterpreted. In 12 variants (12/84, 14.3%), classification changed from (likely) pathogenic to variant of unknown significance (VUS). One of these variants was a hypomorphic allele escaping the standard variant classification. Individuals with variants that downgraded to VUS after reevaluation showed significantly lower Schwartz scores and QTc intervals compared to individuals with unchanged variant characterization.

**Conclusion:**

This finding confirms genetic variant interpretation as a dynamic process and underlines the importance of ongoing genetic counseling, especially in LQTS patients with minor clinical criteria.

## Graphical abstract

We aimed to evaluate the rate of change in variant classification based on current interpretation standards and dependent on clinical findings in children with LQTS. In 12 out of 84 reinterpreted variants (14.3%), classification changed from (likely) pathogenic to variant of unknown significance (VUS). Individuals with variants that downgraded to VUS after reevaluation showed significantly lower Schwartz scores and QTc intervals compared to individuals with unchanged variant characterization.

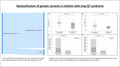

## 1 INTRODUCTION

Long QT syndrome (LQTS) is a cardiac arrhythmia disorder characterized by prolonged QT‐interval on ECG and increased risk for life‐threatening arrhythmias. The inherited form of LQTS is classified as channelopathy with an estimated prevalence of 1:2,500 (Lieve & Wilde, 2015; Schwartz et al., 2009). Clinical symptoms might present as syncopal events caused by tachyarrhythmia, especially ventricular tachycardia (*torsades de pointes*). Ventricular tachycardia might terminate without further intervention or can turn into ventricular fibrillation leading to sudden cardiac death (Alders, Bikker, & Christiaans, 1993). Preventative measures in patients with LQTS consist in lifestyle modification (avoidance of triggers) and beta‐blocker therapy (Priori et al., 2013). For patients with cardiac events despite the intake of beta‐blockers or with intolerance to beta‐blockers, implantation of a cardioverter–defibrillator might be indicated (Alders et al., 1993; Writing Committee, 2017). Several subtypes of LQTS have been described so far, differing from each other based on underlying protein defect, distinct QT appearance, and different triggers for cardiac events (Alders et al., 1993). The different subgroups of LQTS are caused by variants in distinct genes (Bohnen et al., 2017) with most of these variants located in either *KCNQ1* (MIM *607542, LQTS 1), *KCNH2* (MIM *152427, LQTS 2), or *SCN5A* (MIM *600163, LQTS 3) (Giudicessi & Ackerman, 2013; Schwartz, Crotti, & Insolia, 2012), coding for potassium or sodium ion channels, respectively. It should be noted that some of the reported LQTS‐associated genes have recently been doubted to be causative for LQTS in a monogenic context (Adler et al., 2020).

A causative variant can be detected in about 80% of cases with inherited LQTS (Giudicessi & Ackerman, 2013; Schwartz et al., 2012), but there is a lack of updated studies concerning the diagnostic yield, specifically in children. Sequence interpretation remains challenging despite a growing number of data analysis platforms and major advances in molecular technologies. Standards on interpretation and classification of variants published by the American College of Medical Genetics and Genomics (ACMG) in 2015 (Richards et al., 2015) are widely used today. Those guidelines recommend the use of specific standard terminology and classification of variants into five categories based on distinct criteria, such as clinical phenotype, population data, computational data, functional data, and segregation data. Pathogenic (class V) and likely pathogenic (class IV) variants are considered disease causing. Variants of unknown significance (class III) need to be interpreted in the individual setting and (likely) benign (class I, II) variants are unlikely to be associated with the disease. There is a limitation of applying ACMG recommendations to the interpretation of LQTS‐associated variants that do not fit in the classical concept of monogenic inherited diseases (Giudicessi, Roden, Wilde, & Ackerman, 2018).

Open genetic databases, such as the genome aggregation database (gnomAD) or the ClinVar database, helped to improve variant interpretation over time. However, variant interpretation is not only dependent on the date of report, but can also differ between the performing laboratories, because of private information. Also, complex pathophysiology makes it hard to predict the functional consequence of a detected variant in a LQTS‐associated gene solely on its DNA location and modification. Due to the consequences in case of carrier‐status of pathogenic variants in one of the known disease genes, such as psychological impact and preventive therapy with lifestyle modification and beta‐blocker therapy, it is of utmost importance to keep variant interpretation updated.

The aim of this study was to evaluate the change in classification of previously reported LQTS‐causative variants after reinterpretation according to current ACMG guidelines and its dependency on clinical findings.

## 2 MATERIAL AND METHODS

### 2.1 Ethical compliance

The study was performed according to the declaration of Helsinki and the STROBE guidelines and approved by the local ethic committee (Study number 243/17S). All parents gave written informed consent for retrospective analysis of data.

### 2.2 Study design

Medical charts from 127 patients (102 families) with a molecular genetic diagnosis of LQTS presented at the German heart center in Munich (*N* = 82) and Leipzig (*N* = 45) between the age of 0 and 18 years were reviewed. Diagnostic molecular genetic testing was performed in patients with a clinical diagnosis or suspicion of LQTS. Predictive molecular genetic testing was performed in patients with an affected relative and a known (likely) pathogenic variant. The variants were originally interpreted and reported in the time between December 2001 and November 2018 (Table S1). Reinterpretation was performed in March 2019.

### 2.3 Variant interpretation

Variant interpretation was performed according to the standards and guidelines for the interpretation of sequence variants published by the ACMG (Richards et al., 2015). Recommendations for interpreting LoF variants (Abou Tayoun et al., 2018) were additionally used. In brief, information about variants were obtained from databases, such as the ClinVar database (https://www.ncbi.nlm.nih.gov/clinvar/) and the Human Gene Mutation Database (HGMD^®^) Professional. Additional PubMed based (https://www.ncbi.nlm.nih.gov/pubmed) literature review was performed when needed. In silico prediction scores, such as CADD (https://cadd.gs.washington.edu/snv), SIFT (https://sift.bii.a‐star.edu.sg/), and PolyPhen‐2 (http://genetics.bwh.harvard.edu/pph2/), were used for evaluating computational evidence. Amino acid conservation was assessed by using the UCSC genome browser (https://genome.ucsc.edu/). The gnomAD dataset (https://gnomad.broadinstitute.org/) was used as controls. Gene constraints from gnomAD were utilized for assessing the predicted impact of missense variants on genes. Variant location in functional domains was reviewed by using the UniProt dataset (https://www.uniprot.org/). For KCNQ1, the transmembrane/linker/pore region (amino acid 122–348) and the C‐terminus were considered as functional domains (Kapa et al., 2009). Hotspots in the C‐terminus were specified as three specific regions (amino acid 349–391, 509–575, 585–607; Kapplinger et al., 2015). Since it is known that heterozygous mutations in the pore region of KCNH2 (amino acid 548–659) have a strong dominant‐negative effect (Anderson et al., 2014), that region was considered as hot spot. In SCN5A, the four domains (amino acid 113–420, 699–969, 1,187–1,501, 1,510–1,807) of which each includes six transmembrane segments (S1–S6; Payandeh, Scheuer, Zheng, & Catterall, 2011) were expected to be functional regions (Kapa et al., 2009). The ACMG criteria were applied by using the bioinformatics software tool InterVar (http://wintervar.wglab.org/). The following GeneBank reference sequences were used for the studied genes: *KCNQ1* (NM_000218.2), *KCNH2* (NM_000238.3), *SCN5A* (NM_198056.2), *KCNJ2* (NM_000891.2), *CALM2* (NM_001743.5).

### 2.4 Clinical data

Clinical data relevant to the diagnosis of LQT were recorded from medical charts. The Schwartz score was used as a scoring system for clinical diagnosis of LQTS, based on ECG variables (QTc, presence of ventricular tachycardias, abnormal T‐waves, bradycardia), a clinical history of syncope, and a family history of LQTS or sudden cardiac death (SCD; Schwartz & Crotti, 2011). Clinical parameters were compared between the group of patients in whom the previously reported (likely) pathogenic variant remained (likely) pathogenic and those in whom reinterpretation reclassified a previously reported (likely) pathogenic variant to a variant of unknown significance (VUS). Major arrhythmic events (MAEs) were defined as occurrence of SCD, aborted SCD, appropriate implantable cardioverter–defibrillator discharge or sustained ventricular tachycardia.

### 2.5 Statistics

Statistical analysis was performed with the SPSS software version 25.0.0 (SPSS Inc.; IBM Company). Based on the small study population normal distribution of variables could not be assumed and only nonparametric tests were used. Continuous variables are expressed as median [minimum–maximum] and were analyzed by Wilcoxon rank sum test when analyzed between the groups. Categorical variables are given as percentages of group totals and were analyzed by Pearson's chi square or Fisher's exact tests, where appropriate. A *p*‐value of less than .05 (two‐sided) was considered statistically significant.

## 3 RESULTS

### 3.1 The distribution of LQTS‐causing variants and genes in this study

There was a total of 84 different variants originally reported as disease causing (pathogenic or likely pathogenic) in 127 children from 102 families. Most variants were in genes encoding for cardiomyocyte potassium channels, followed by genes encoding for sodium channel and calcium handling proteins (Table 1). More than two‐thirds of variants were missense; potential loss of function variants, that is, frameshift, nonsense, splice, nearsplice variants, and gross intragenic deletions, reached a proportion of 29.8% (25/84). Almost all reclassified variants were missense variants (91.7%, 11/12; Table 2).

**Table 1:** Distribution of LQTS‐associated genes in this study

Gene	Total, *N* = 84	Unchanged (likely) pathogenic, *N* = 72	Reclassified VUS, *N* = 12	*p*‐value^a^
*KCNQ1*	50.0% (42/84)	54.2% (39/72)	25% (3/12)	.12
*KCNH2*	36.9% (31/84)	33.3% (24/72)	58.3% (7/12)	.12
*SCN5A*	9.5% (8/84)	8.3% (6/72)	16.7% (2/12)	.32
*KCNJ2*	2.4% (2/84)	2.8% (2/72)	0% (0/12)	NA
*CALM2*	1.2% (1/84)	1.4% (1/72)	0% (0/12)	NA

Abbreviations: LQTS, long QT syndrome; NA, not available; VUS, variant of unknown significance.

^a^ Fisher's exact test.

**Table 2:** Distribution of reclassified variants in this study

Variant	Total, *N* = 84	Unchanged (likely) pathogenic, *N* = 72	Reclassified VUS, *N* = 12	*p*‐value^a^
Missense, *N*/*n* (%)	67.9% (57/84)	63.9% (46/72)	91.7% (11/12)	.092
Frameshift, *N*/*n* (%)	15.5% (13/84)	18.1% (13/72)	0% (0/12)	NA
Nonsense, *N*/*n* (%)	7.1% (6/84)	8.3% (6/72)	0% (0/12)	NA
Splice	3.6% (3/84)	4.2% (3/72)	0% (0/12)	NA
Small inframe deletion/duplication	2.4% (2/84)	2.8% (2/72)	0% (0/12)	NA
Nearsplice	2.4% (2/84)	1.4% (1/72)	8.3% (1/12)	.267
Intragenic deletion	1.2% (1/84)	1.4% (1/72)	0% (0/12)	NA

Abbreviations: NA, not available; VUS, variant of unknown significance.

^a^ Fisher's exact test.

### 3.2 Change of variant classification after reinterpretation

When applying the ACMG criteria to the variants originally reported as LQTS‐causing (pathogenic or likely pathogenic), classification changed to variant of unknown significance (VUS) in 14.3% (12/84) of cases (Table 3). Those 12 variants affected a total of 19 patients.

**Table 3:** List of variants of which the classification changed from (likely) pathogenic to VUS after reevaluation according to ACMG guidelines

Gene	Variant	Predicted functional consequence	Applied ACMG criteria	ACMG classification	Date of last report
*KCNQ1* NM_000218.2	c.1109C>T, p.(Ala370Val)	Missense	PM1, PP5, BS1	VUS	02.07.2011
*KCNQ1* NM_000218.2	c.1261A>G, p.(Lys421Glu)	Missense	PM2	VUS	01.11.2005
*KCNQ1* NM_000218.2	c.2018A>G, p.(Asp673Gly)	Missense	PM2	VUS	21.07.2014
*KCNH2* NM_000238.3	c.526C>T, p.(Arg176Trp)	Missense	PS3, PS4, PP2, PP3, PP5, BS1, BS3, BP6	VUS^a^	15.02.2018
*KCNH2* NM_000238.3	c.775G>A, p.Asp259Asn	Missense	PM2, PP2, PP5	VUS	29.08.2006
*KCNH2* NM_000238.3	c.1178C>T, p.(Pro393Leu)	Missense	PM2, PP2, PP3, PP5	VUS^b^	20.03.2018
*KCNH2* NM_000238.3	c.1225G>T, p.(Val409Leu)	Missense	PM2, PP2, PP3	VUS	24.01.2012
*KCNH2* NM_000238.3	c.2090T>A, p.(Leu697His)	Missense	PM2, PP2, PP3, PP1	VUS^b^	01.07.2012
*KCNH2* NM_000238.3	c.2588G>A, p.(Arg863Gln)	Missense	PM2, PP3, PP2, PP5	VUS^b^	08.06.2009
*KCNH2* NM_000238.3	c.2948C>T, p.(Thr983Ile)	Missense	PP2, PP3, PP5, BS1	VUS	17.01.2012
*SCN5A* NM_198056.2	c.536G>A, p.(Arg179Gln)	Missense	PM1, PM2, PP3	VUS	14.08.2013
*SCN5A* NM_198056.2	c.998+5G>A, p.(?)	Nearsplice	PM1, PP3, BS1	VUS	24.09.2015

Abbreviations: VUS, variant of unknown significance.

^a^ The variant should be considered as a complex variant, that is, with disease modifying influence.

^b^ Classification of these variants as VUS depended on the applied criterion PP4 (see text for further detail).

If the phenotype of a patient was characteristic for causative variants in one single gene and a variant was identified in this gene, PP4 was applied. In context of LQTS, phenotype was assessed by specific morphologies of the ST‐segment and T‐wave on surface ECG. In three variants that were identified in four different individuals (patient IDs 19, 110, 116, 2141), the application of PP4 was critical for the classification as either “VUS” or “likely pathogenic” (Table 2). ECG morphology was nonspecific for a LQTS subtype or gene in three of those four patients (patient IDs 19, 110, 116). One patient's ECG (patient ID 2,141) showed broad‐based T‐waves typically found in LQT type 1 but the identified variant was located in *KCNH2* associated with LQT2.

Although half of all variants were located in *KCNQ1*, most of the VUS after reclassification were located in *KCNH2* (58.3%, 7/12, *p* = .12). No reclassification occurred in previously identified (likely) pathogenic variants in the *KCNJ2* (MIM *600681) or *CALM2* (MIM *114182) gene (Table 1), with both carriers of pathogenic variants in *KCNJ2* being syndromic cases with Andersen–Tawil syndrome. Most of the reclassified variants were missense variants (11/12, 91.7%, *p* = .092; Table 2). Out of all 57 missense variants, 19.3% were reclassified as VUS.

### 3.3 Comparison of clinical characteristics

**Figure 1:**
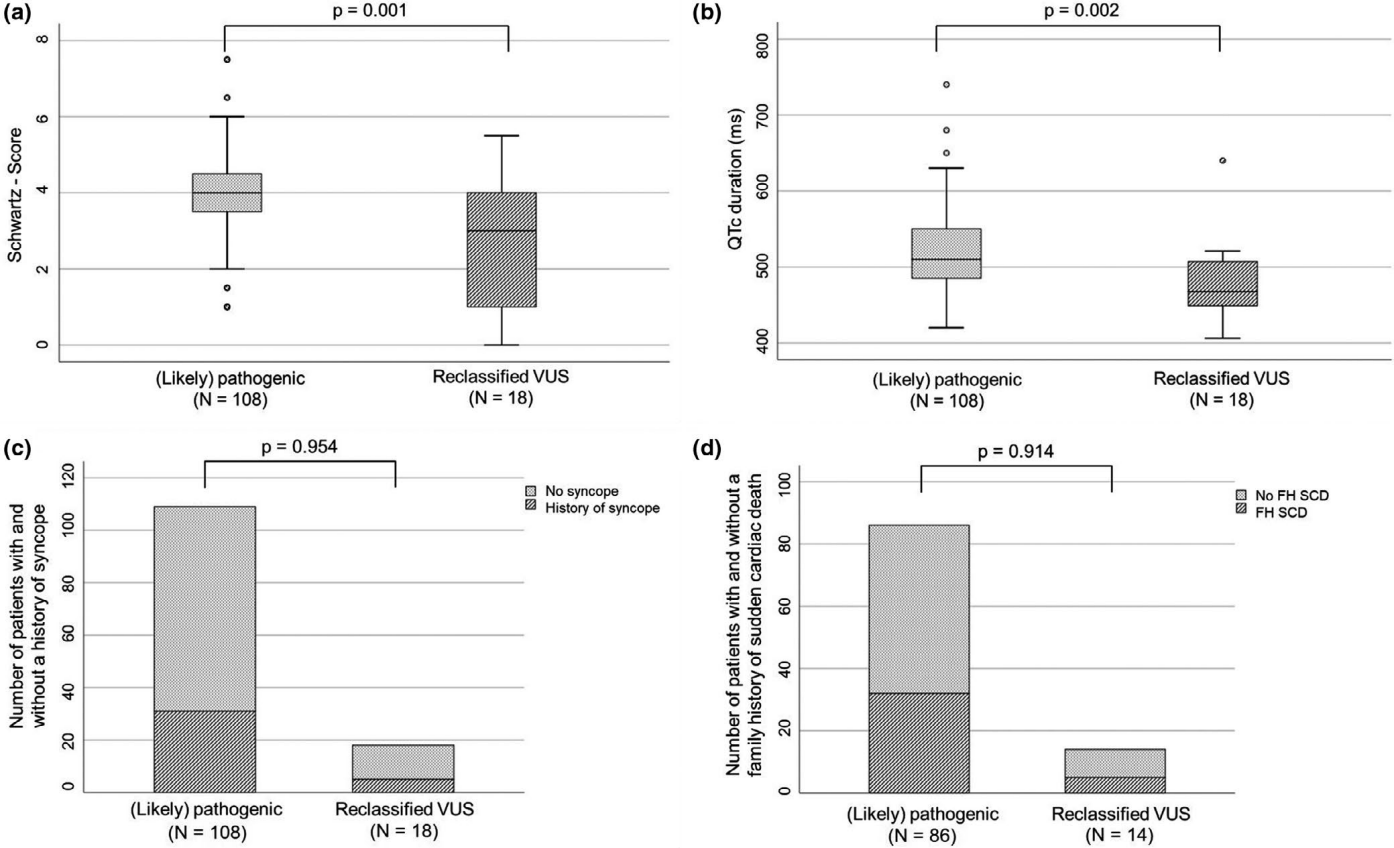
The Schwartz score, a scoring system used to help establishing the clinical diagnosis of long QT syndrome in a patient (Schwartz & Crotti, 2011; panel a) and the maximum measured QTc time (panel b) were significantly lower in patients in whom variants were reclassified from (likely) pathogenic to variant of unknown significance (VUS). There was no significant difference between groups in percentage of patients with and without syncope (panel c) and with and without a family history of sudden cardiac death (panel d); information for the family history was not available in all patients; FH, family history; SCD, sudden cardiac death

Clinical characteristics of the 18 children in whom the genetic variant changed from originally reported (likely) pathogenic to VUS after reinterpretation were compared to the clinical findings of the remaining 108 patients in whom the genetic variant remained disease causing after reinterpretation (Table 3). One individual fell in both categories and was excluded, because of carrying two different variants classified as either VUS or pathogenic after reevaluation (patient ID 2102). There was no difference between the reclassified VUS group of patients compared to the nonreclassified group of patients with regard to gender, age at diagnosis, age at genetic testing, and follow‐up time. Change in variant pathogenicity occurred more often in LQT2 patients and less often in LQT1 and LQT3 patients (Table 1). The Schwartz score (Schwartz & Crotti, 2011) was lower (Figure 1, panel a) and maximal QTc time was shorter (Figure 1, panel b) in patients with a VUS after reevaluation compared to patients with unchanged (likely) pathogenic variants. There was no statistically significant difference in history of syncope (Figure 1, panel c), family history of SCD (Figure 1, panel d), or the occurrence of major arrhythmic events between the VUS cohort and the unchanged (likely) pathogenic variant cohort (Table 4). Excluding patients from this analysis who carried either a homozygous variant, compound‐heterozygous variants, a second (likely) pathogenic variant in another LQTS causing gene or patients who were affected by syndromic forms of LQTS (i.e., Andersen–Tawil syndrome, Jervell and Lange–Nielsen syndrome) had no effect on significant differences.

**Table 4:** Clinical characteristics of patients

Characteristics	Total, *N* = 126	(Likely) pathogenic, *N* = 108	Reclassified VUS, *N* = 18	*p*‐value^a^
Male, *N*/*n* (%)	63/126 (50)	51/108 (47.2)	11/18 (61.1)	.203^c^
Age at diagnosis (years), median [range]	7.7 [0.0–18.0]	7.7 [0.0–18.0]	7.7 [0.0–16.2]	.989^d^
Age at genetic testing (years), median [range]	9.3 [0.0–26.2]	9.9 [0.0–26.3]	9.3 [0.2–16.5]	.48^d^
Follow‐up time (years), median [range]	4.6 [0.1–24.3]	4.7 [0.1–24.3]	3.1 [0.4–6.2]	.157^d^
Clinical diagnosis at first encounter				
LQT, *N*/*n* (%)	6/126 (4.8)	6/108 (5.6)	0/18 (0)	.233^c^
LQT1, *N*/*n* (%)	37/126 (29.4)	36/108 (33.3)	1/18 (5.6)	
LQT2, *N*/*n* (%)	34/126 (27.0)	27/108 (25.0)	7/18 (38.9)	
LQT3, *N*/*n* (%)	7/126 (5.6)	5/108 (4.6)	2/18 (11.1)	
LQT7, *N*/*n* (%)	1/126 (0.8)	1/108 (0.9)	0/18 (0)	
PVBs, *N*/*n* (%)	1/126 (0.8)	1/108 (0.9)	0/18 (0)	
Syncope of unknown etiology, *N*/*n* (%)	1/126 (0.8)	1/108 (0.9)	0/18 (0)	
Differential diagnosis LQT, *N*/*n* (%)	39/126 (31.0)	31/108 (28.7)	8/18 (44.4)	
Diagnosis at follow‐up				
LQT, *N*/*n* (%)	5/126 (4.0)	5/108 (4.6)	0/18 (0)	.018^c^
LQT1, *N*/*n* (%)	57/126 (45.2)	54/108 (50.0)	3/18 (16.7)	
LQT2, *N*/*n* (%)	51/126 (40.5)	39/108 (36.1)	12/18 (66.7)	
LQT3, *N*/*n* (%)	11/126 (8.7)	8/108 (7.4)	3/18 (16.7)	
LQT7, *N*/*n* (%)	2/126 (1.6)	2/108 (1.9)	0/18 (0)	
Schwartz Score,^b^ median [range]	4 [0–7.5]	4 [1–7.5]	3 [0–5.5]	.001^d^
Maximal QTc time (ms), median [range]	505 [410–740]	510 [420–740]	470 [410–640]	.001^d^
Family history of SCD in relative,^e^ *N*/*n* (%)	37/100 (37)	32/86 (37.2)	5/14 (35.7)	.914^c^
History of syncope, *N*/*n* (%)	36/126 (28.6)	31/108 (28.7)	5/18 (27.8)	.954^c^
Major arrhythmic events,^f^ *N*/*n* (%)	22/12 (17.5)	20/108 (18.5)	2/18 (11.1)	.452^c^

Abbreviations: *N*/*n*, number of patients; PVBs, premature ventricular beats; SCD, sudden cardiac death; VUS, variant of unknown significance.

^a^ Pathogenic versus reclassified VUS.

^b^ The Schwartz score is a scoring system used to help establishing the clinical diagnosis of long QT syndrome in a patient (Schwartz & Crotti, 2011).

^c^ Chi‐squared‐test.

^d^ Mann–Whitney–Wilcoxon test.

^e^ Information not available in all patients.

^f^ Major arrhythmic event defined as occurrence of sudden cardiac death, aborted sudden cardiac death, appropriate implantable cardioverter–defibrillator discharge or sustained ventricular tachycardia.

## 4 DISCUSSION

To assess the time and laboratory dependent change in variant interpretation, previously reported (likely) pathogenic LQTS variants were reevaluated in 127 children in this study. Variants were reclassified according to the standards and guidelines for the interpretation of sequence variants published by the ACMG 2015 (Richards et al., 2015). The 84 different reinterpreted variants in this study represent the distribution of LQTS‐causing genes reported in the literature with *KCNQ1*, *KCNH2,* and *SCN5A* being the main cause, although variants in *KCNQ1* were overrepresented with 50% (compared to 30%–35% in the literature; Alders et al., 1993). The proportion of missense variants (67.9%) was similar to the reported one in the literature (approximately 70%; Alders et al., 1993). After reinterpretation, a noteworthy proportion of about 14% (12/84) of the variants was reclassified as VUS based on current knowledge. This finding is in line with previous studies that showed downgrading of published LQTS1‐ and LQTS2‐causative missense variants in about 7%–13% of variants (Clemens et al., 2018; Mattivi et al., 2019). That fact is of high significance, specifically in predictive molecular genetic cascade screening of further familiy members. A molecular genetic diagnosis of LQT will impact patients' management and quality of life and misinterpretation can cause harm, specifically in children with this diagnosis.

Variant interpretation according to the current ACMG standards is challenging, specifically in hypomorphic variants that have attenuated effects. The variant c.526C>T, p.(Arg176Trp) in *KCNH2* (NM_000238.2) falls in this category. When strictly applying the ACMG criteria, this variant is classified as VUS. On the one hand, it is a frequent variant (gnomAD: allele frequency: 0.04%) that was classified as likely benign or VUS by different laboratories (ClinVar ID: 67509), also due to functional testing in morpholino knockdown zebrafish (Jou et al., 2013). On the other hand, there are studies that showed that the variant leads to significant QTc interval prolongation (Fodstad et al., 2006) and that it has a functional effect on electrophysiological behavior of cells *in vitro* (Fodstad et al., 2006; Lahti et al., 2012). It is supposed that carrier of this variant are prone to arrhythmia under certain conditions (Lahti et al., 2012; Marjamaa et al., 2009) and that the variant is most likely a hypomorphic allele. In two out of seven patients carrying this variant in our cohort, there was a second LQTS‐associated variant identified (one reclassified VUS in *KCNH2*, one pathogenic variant in *KCNQ1*, Table S1) supporting this assumption. There is a conflict for laboratories in how to report these nonclassic pathogenic variants which was recently addressed by Giudicessi et al. (2018). The authors proposed to expand the ACMG classification by different risk alleles to make variants reportable for laboratories (Giudicessi et al., 2018). Without the hypomorphic variant in *KCNH2*, the proportion of originally reported (likely) pathogenic reclassified as VUS still reaches 13.1% (11/84).

A further challenge in variant interpretation is that the molecular pathophysiology that leads to impaired channel function is often diverse and complex. In case of *KCNQ1*, for example, pathogenic variants affect different molecular pathways and interactions with other proteins (Bohnen et al., 2017) dependent from its location and effect. The knowledge of these complex mechanisms, however, is important for applying the moderate criterion PM1 (variant location in a “mutational hot spot and/or critical and well‐established functional domain”). Another moderate criterion, PM2, can be applied, if a variant is “absent from controls”. Today, the gnomAD dataset helps in estimating allele frequencies with about 140,000 sequenced individuals. However, reduced penetrance in patients carrying pathogenic LQTS‐causing variants (Alders et al., 1993) aggravates interpretation of allele frequencies. Another difficulty in variant interpretation is that a few of the criteria might be executed diversely by different laboratories. In our study, the supporting criterion PP4 was critical for classification in three variants (3/84, 3.6%). Although we did not apply PP4 in those cases after reanalyzing their ECGs, some laboratories might because of characteristic ECGs in their cases.

Variant interpretation is not static but a fluid process that can change in time and makes variant reinterpretation necessary, because of practical consequences for patients and their relatives. Findings of the current study also support the importance of detailed and repeated cardiac examination, as the Schwartz score was significantly reduced in children with variants downgraded to VUS after reinterpretation. Surprisingly, history of syncopes as well as family history of sudden cardiac death was not statistically different between these two compared groups. Especially, family history should be expected to be a strong indicator for an inherited disease. The lack of significance concerning the family history, however, might be explained by the known reduced penetrance of LQTS (Goldenberg et al., 2011; Priori et al., 2003) or by the relatively small group in this study. Nevertheless, our study shows that the Schwartz score, which is calculated from all other three parameters (QTc duration, history of syncopes, family history; Schwartz & Crotti, 2011), is a solid clinical tool when it comes to evaluate the probability of a definite diagnosis of LQTS restricted by the fact that this tendency might not be helpful in diagnosis of a single case.

In conclusion, our study quantifies the necessity of variant reinterpretation by outlining the notable amount of about 14% in change of variant classification. This supports the importance of appropriate counseling of patients undergoing molecular genetic testing and the need for regular genetic recounseling during follow‐up. When making the diagnosis of LQT, clinicians should be aware of the possibility of reclassification, specifically in children with lower clinical suspicion.

### 4.1 Limitations

The usual limitations of a retrospective study apply to this analysis. Distinct accredited laboratories contributed to molecular genetic diagnosis and the reports most of the time did not mention the genetic panels behind used for DNA analysis. Therefore, an unidentified pathogenic variant in another, not tested LQTS‐associated gene cannot be excluded. Furthermore, the group of patients with variants that were reclassified to VUS was small with only 19 individuals. Therefore, findings regarding comparison of both groups should be considered with caution.

## CONFLICT OF INTEREST

The authors report no competing interests.

## AUTHOR CONTRIBUTIONS

Study design: DSW, TB and CW. Drafting the manuscript: DSW, TB and CW. Obtaining clinical data: TB, RG, GH and CW. Genetic analysis: DSW and AM‐G. Critical reviewing the manuscript and final approval: all.

## Supporting information

Table S1

## Data Availability

The data that support the findings of this study are available from the corresponding author upon reasonable request.
